# Prenatal retinoic acid exposure reveals candidate genes for craniofacial disorders

**DOI:** 10.1038/s41598-018-35681-0

**Published:** 2018-11-30

**Authors:** Marie Berenguer, Muriel Darnaudery, Stéphane Claverol, Marc Bonneu, Didier Lacombe, Caroline Rooryck

**Affiliations:** 1 University Bordeaux, Maladies Rares: Génétique et Métabolisme (MRGM), U 1211 INSERM, F-33000 Bordeaux, France; 2 Université de Bordeaux, Nutrition et neurobiologie intégrée (NUTRINEURO), UMR 1286, 146, rue Léo Saignat, 33076 Bordeaux Cedex, France - Inra, Nutrition et neurobiologie intégrée (NUTRINEURO), UMR 1286, F-33076 Bordeaux, France; 3 Center of Functional Genomics, Bordeaux University, Bordeaux, France; 4 CHU de Bordeaux, Service de Génétique Médicale, Centre de Référence Anomalies du Développement et Syndromes Malformatifs, F-33000 Bordeaux, France

## Abstract

Syndromes that display craniofacial anomalies comprise a major class of birth defects. Both genetic and environmental factors, including prenatal retinoic acid (RA) exposure, have been associated with these syndromes. While next generation sequencing has allowed the discovery of new genes implicated in these syndromes, some are still poorly characterized such as Oculo-Auriculo-Vertebral Spectrum (OAVS). Due to the lack of clear diagnosis for patients, developing new strategies to identify novel genes involved in these syndromes is warranted. Thus, our study aimed to explore the link between genetic and environmental factors. Owing to a similar phenotype of OAVS reported after gestational RA exposures in humans and animals, we explored RA targets in a craniofacial developmental context to reveal new candidate genes for these related disorders. Using a proteomics approach, we detected 553 dysregulated proteins in the head region of mouse embryos following their exposure to prenatal RA treatment. This novel proteomic approach implicates changes in proteins that are critical for cell survival/apoptosis and cellular metabolism which could ultimately lead to the observed phenotype. We also identified potential molecular links between three major environmental factors known to contribute to craniofacial defects including maternal diabetes, prenatal hypoxia and RA exposure. Understanding these links could help reveal common key pathogenic mechanisms leading to craniofacial disorders. Using both *in vitro* and *in vivo* approaches, this work identified two new RA targets, Gnai3 and Eftud2, proteins known to be involved in craniofacial disorders, highlighting the power of this proteomic approach to uncover new genes whose dysregulation leads to craniofacial defects.

## Introduction

Craniofacial anomalies are one of the most frequent birth defects observed^1^ and more than 700 disorders are associated with craniofacial features in OMIM database (https://www.ncbi.nlm.nih.gov/omim). Among them, some are now well characterized phenotypically and genetically, such as Treacher Collins Syndrome (TCS, MIM: #154500). Indeed, pathogenic variants have been identified in *TCOF1*, *POLR1D*, and *POLR1C* genes in patients presenting with bilateral mandibular and malar hypoplasia, downslanting palpebral fissures, and microtia^2^. Moreover, one of the underlying molecular mechanisms of this syndrome has been elucidated by identifying a link with p53 signaling and apoptosis function^3,4^. Unfortunately, not all craniofacial disorders have yet been characterized on molecular level. As an example, Oculo-Auriculo-Vertebral Spectrum (OAVS) is a developmental disorder involving, like TCS, the two first branchial arch derivatives. Patients typically present with ear anomalies (microtia/anotia, preauricular tags, ear dysplasia), eye defects (epibulbar dermoid, microphtalmia or eyelid coloboma), vertebral abnormalities (hemivertebrae, vertebral puzzle), associated with hemifacial microsomia as well as cardiac and renal anomalies^5,6^. OAVS has been associated with both environmental factors such as retinoic acid (RA) exposure^7^ and genetic causes including numerous copy number variations (CNVs)^7–9^. However, recurrent CNVs were rarely identified, thus failing to identify a major causative locus or gene. Whole exome sequencing performed on selected cases recently led to the identification of *MYT1* (NM_004535.2) as the first gene involved in OAVS^6^, but the low frequency of variants identified in this gene among the different cohorts (3/226)^6,10^ prompted us to look for new causative genes.

Several environmental factors had also been associated with craniofacial disorders such as maternal diabetes^11,12^, hypoxia or vascular disruption^13–15^ and teratogenic agent exposures^16,17^. Among them, acute vitamin A intoxication or RA exposure during pregnancy were correlated with a high risk of spontaneous abortions or major malformations in humans^18^. RA intoxication leads to various craniofacial abnormalities such as microtia/anotia and micrognathia present in various disorders such as TCS or OAVS^18–21^. Experimental studies in animals have analyzed further the dose- and timing-dependent embryonic developmental defects following gestational RA treatment^22–25^. Both visceral and skeletal anomalies have been observed in various species and notably, RA administration at E9.5 led to hypoplasia of the branchial arches (79%), as well as auricular (47%) and eye (12.5%) anomalies in mice^26^. Additionally, an increase of RA signaling in chicken embryos treated by an inhibitor of Cyp26 enzymes involved in RA degradation, led to Di-George syndrome phenocopies^27^, including cardiac and craniofacial abnormalities^28^.

Both vitamin A deficiency^29^ and specific gene knockout (KO) studies^30^ have proven the critical role of RA signaling in several developmental processes^31^. More precisely, all-*trans*-RA (ATRA) is a ligand for the RA receptor (RAR) which forms a heterodimer with another nuclear receptor known as retinoid X receptor (RXR). This complex binds DNA and induces changes in gene expression^32^ during embryonic development^33–35^. In particular, ATRA embryonic exposure induced craniofacial skeletal defects related to *Fgf8* and homeobox gene dysregulation^36^. Additionally, *TBX1*, one of the main human genes involved in DiGeorge syndrome’s phenotype has been shown to be repressed by RA in the zebrafish pharyngeal arches^37^. Finally, our previous studies^6,10^ have highlighted that *MYT1*, the unique OAVS gene identified so far, was linked to the RA signaling pathway.

In the present study, we used a toxicological approach by treating mouse embryos with ATRA to identify new candidate genes and pathways involved in poorly characterized craniofacial disorders. Gestational ATRA treatment at 100 mg/kg led to dysregulation of proteins and modulated some pathways. Success of this approach was reinforced by the identification of proteins known to be involved in craniofacial development and especially of two related to genes already associated in mandibulofacial dystosis. Moreover, we revealed here a link between the major OAVS environmental factors (i.e. ATRA exposure^18,24^, hypoxia^15,38^ and diabetes^11,12^) and craniofacial defects.

## Results

### Prenatal ATRA exposure increases embryonic resorption and leads to craniofacial defects

**Figure 1:**
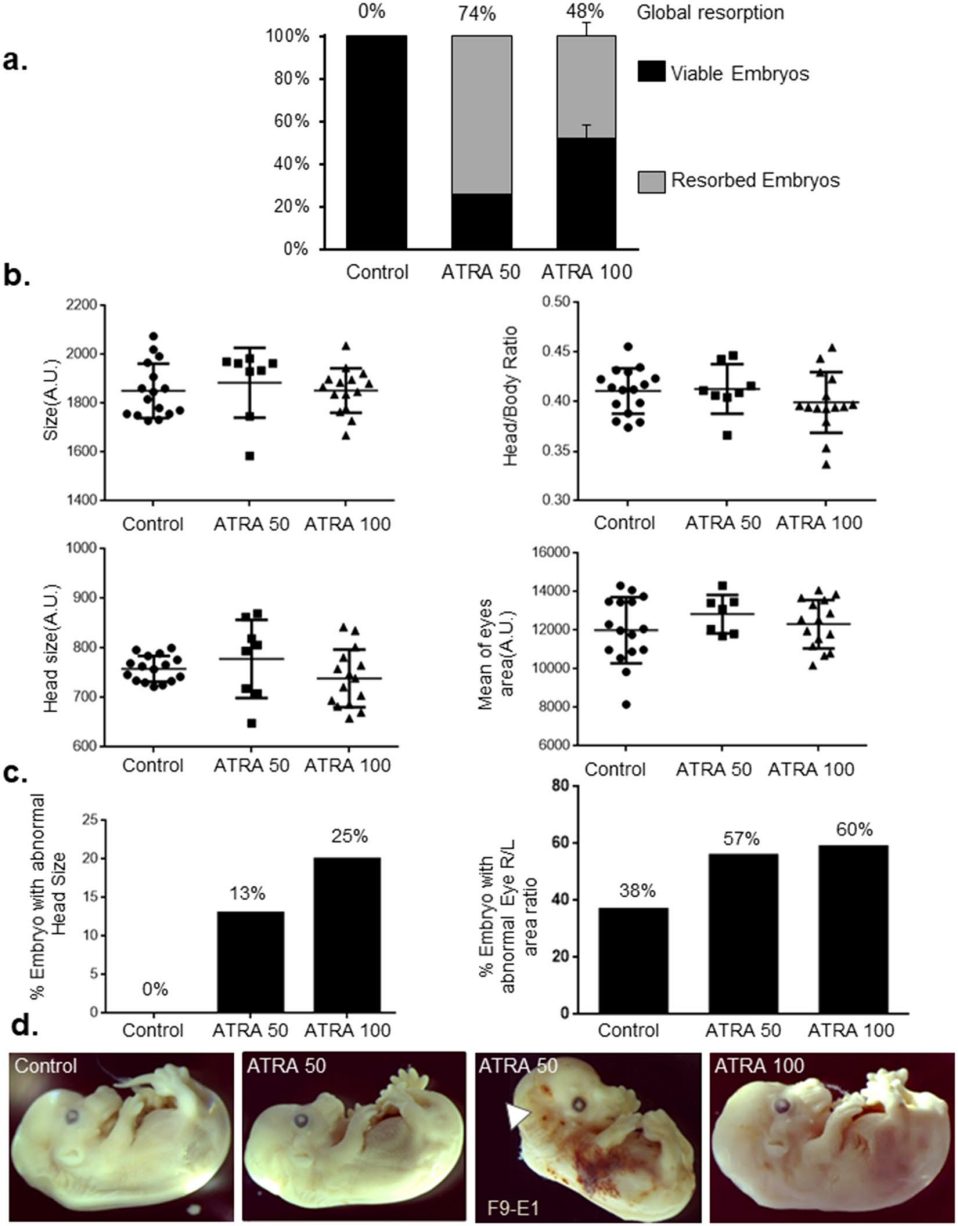
(**a**) Analysis of the embryonic resorption after E9.5 intraperitoneal mice injection of ATRA at 50 mg/kg (one experiment), at 100 mg/kg and control condition (DMSO) (2 independent experiments). Viable embryos are represented in black bars whereas resorbed embryos are in gray bars. (**b**) Head, eye and body size measurements of treated and control embryos at E14.5. No statistical differences were observed concerning global parameters such as embryo size, head size, ratio of size of head and body, area of eyes. (**c**) Percentage of embryos presenting abnormal Head size (left) and eyes area ratio (right). The 90% confidence interval on the mean was computed for each group. Each embryo with a measurement outside the 90% confidence interval was considered with abnormal item. The percentage of embryos with an abnormal head size or abnormal eyes area ratio is calculated (right eye area divided by the left eye area). (**d**) Representative embryos per condition at E14.5. Treated embryos were not, at the first look, especially malformed compared to controls even if hemorrhages have been observed most often in treated embryos. Notably, a bilateral anotia (white arrow) is observed in F9-E1 embryo (ATRA-50). This embryo was excluded of quantitative analysis due to absence of several craniofacial structures (ear, Meckel’s cartilage). n = 16 embryos from 2 females were analyzed for the control group, n = 8 embryos from one female for ATRA 50 and n = 15 embryos from 2 females for ATRA 100.

We examined the impact of ATRA exposure at E9.5 which constitutes the critical period for branchial arch development in mice. Following 50 mg/kg and 100 mg/kg ATRA intraperitoneal injections at E9.5, resorption was observed in 74% and 48% of treated embryos respectively, compared to 0% in control embryos treated with a solution containing the same concentration of DMSO (Fig. 1a). Head, eyes and body size measurements of treated embryos at E14.5 did not reveal any major differences compared to controls (Fig. 1b). However, the proportion of embryos with abnormal head size (measurement outside the 90% confidence interval) and asymmetric eyes area (eye Right/Left area ratio) was increased after ATRA exposure (Fig. 1c). Especially, ATRA treatment may lead to both microcephaly and macrocephaly (Fig. 1b,c) and these defects are observed respectively in 8% and 5% of OAVS patients according two independent cohorts^9,39^. Moreover, even if no statistical analysis could be performed, it seems interesting to mention one particular embryo presenting with a strong phenotype: a bilateral anotia was observed in this only embryo alive among its litter following ATRA 50 treatment (Fig. 1d, white arrow). Interestingly, ear anomalies were the most frequent defects observed following RA exposure in human^18^ and anotia was the most severe ear defect found among OAVS cohorts^6^. Of note, superficial hemorrhages were noticed in several embryos (Fig. 1d).

**Figure 2:**
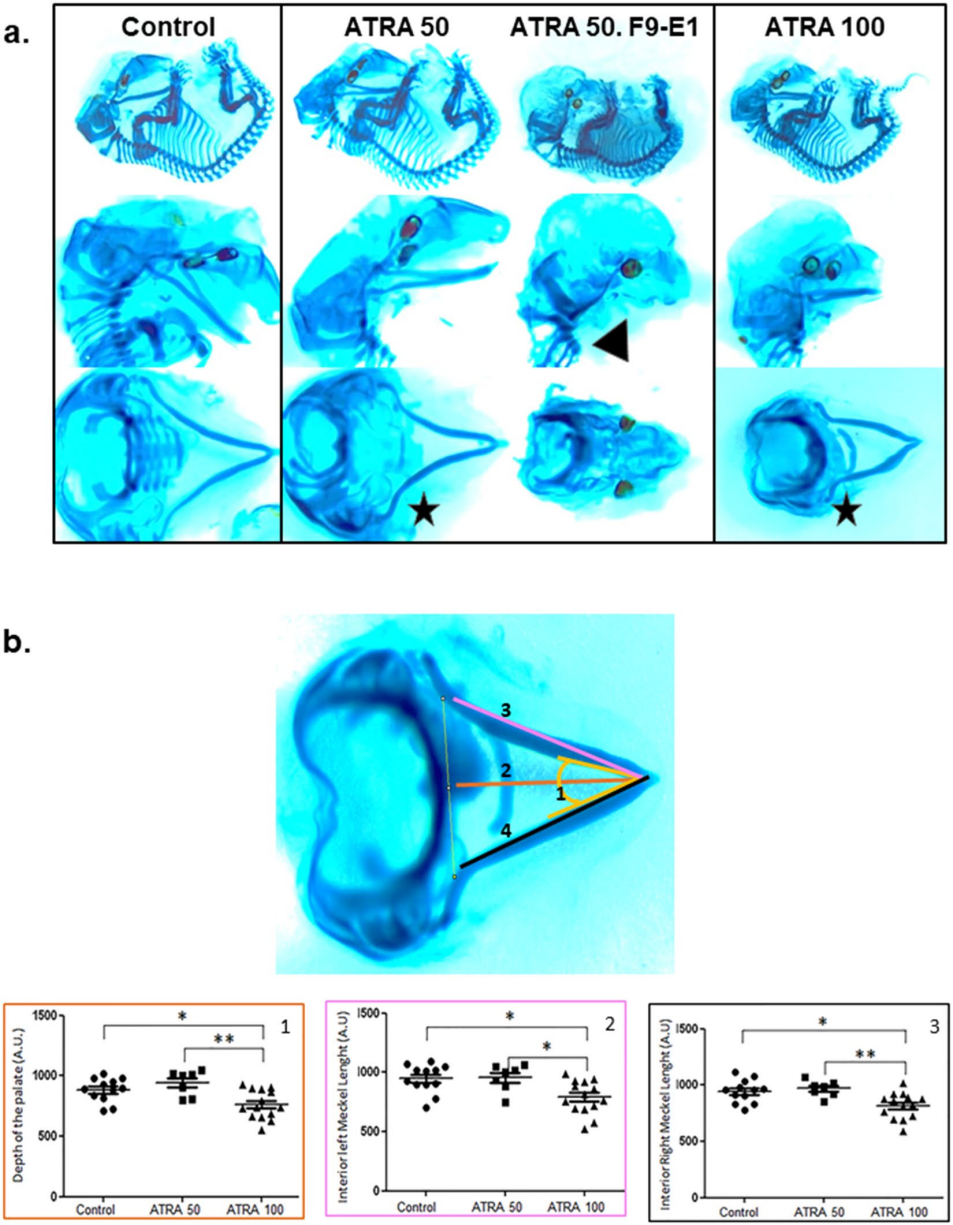
(**a**) Craniofacial cartilages of embryo staining with Alcian Blue-Alizarin Red coloration. Differences in size and/or angle of the Meckel’s cartilage were observed (black stars) after ATRA treatment (50 and 100 mg/Kg). The F9-E1 embryo (ATRA 50) presented a total aplasia of this cartilage (black arrow). This embryo was excluded of quantitative analysis due to absence of several craniofacial structures. (**b**) Quantitative analysis of several craniofacial cartilage parameters. The localization of these 4 different parameters was detailed in Alcian Blue-Alizarin Red staining (up-left panel). ANOVA revealed significant group effect for the depth of the palate (1), F(2) = 6.136 with p = 0.0058; the left length (2) and right (3) length of the Meckel’s cartilage and also, F(3) = 6.453with p = 0.0047; (F(4) = 7.264 with p = 0.0027; Post-hoc analysis by Tukey *p < 0.05, **p < 0.01, ***p < 0.001 were then performed. For Control, n = 16 embryos from 2 females were analyzed, n = 8 embryos from one female for ATRA 50 and n = 15 embryos from 2 females for ATRA-100). For ATRA 100 treatment, the studied parameters are significantly different from control, and then may reveal a real macroscopic effect.

Alcian Blue and Red Alizarin colorations allowed the identification of craniofacial defects in E14.5 ATRA-treated embryos (Fig. 2a, black stars). One-way Anova revealed a significant group effect for each parameter (p = 0.0058 for the depth of the palate; p = 0.0047 and p = 0.0027 for the left and right-side length of the Meckel cartilage respectively). Post-hoc, comparison by Tukey test highlighted that, compared to control embryos, the length of the Meckel cartilage was reduced in ATRA-100 treated embryos: 16.5% smaller in the left size (p = 0.0149) and 13.5% smaller in the right size (p = 0.01); the depth of the palate was also found decreased by 14% (p = 0.0101) in ATRA-100 treated embryos compared to controls. Additionally, it should be noted that a total aplasia of Meckel’s cartilage was observed in the embryo presenting with the bilateral anotia (Fig. 2a, black arrow).

### Prenatal ATRA exposure leads to a dysregulation of proteins involved in metabolic processes, hypoxia pathway and cell-survival signaling

**Figure 3:**
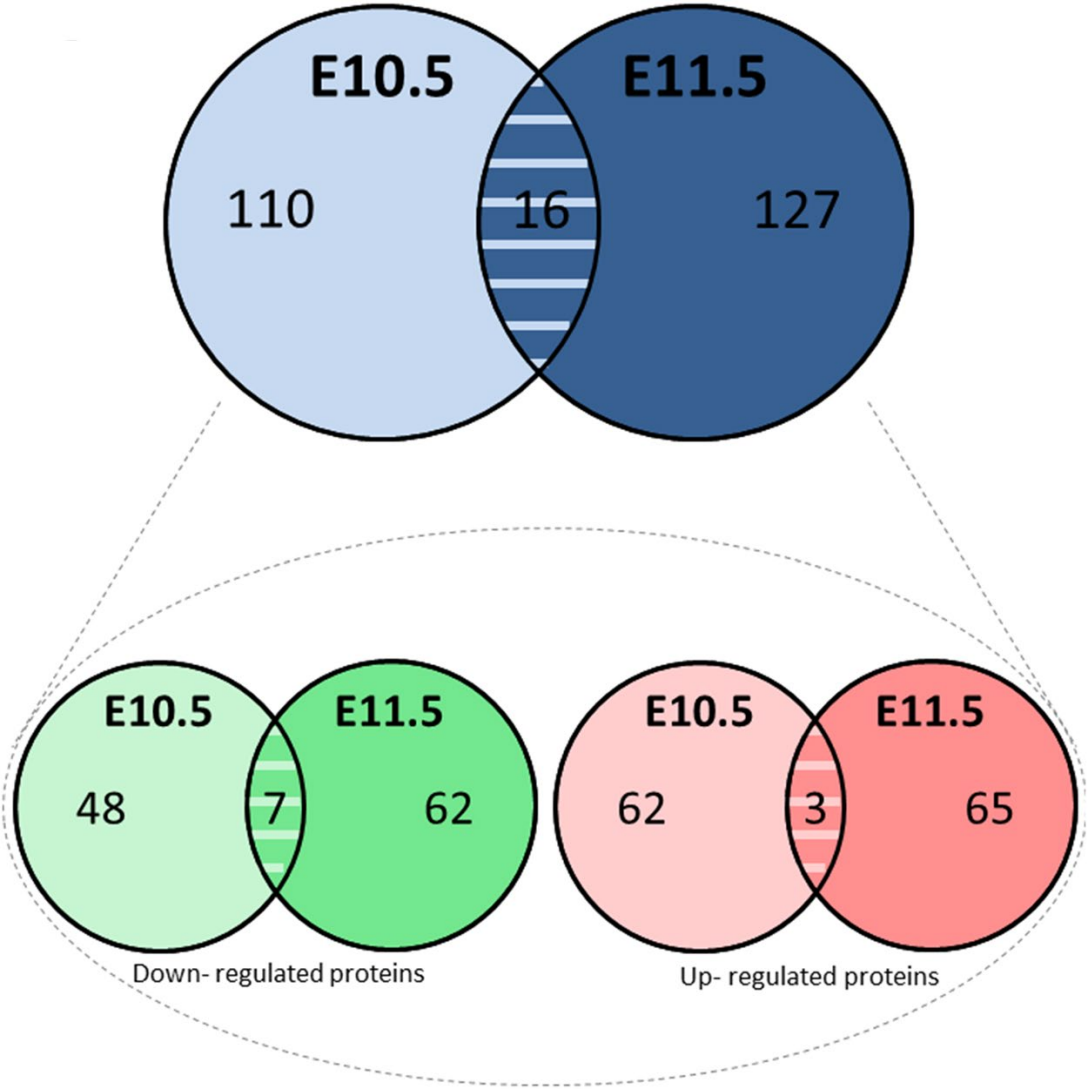
Number of differentially expressed proteins in E10.5 and E11.5 embryos’ heads, identified by LC-MS/MS, following antenatal E9.5 ATRA (100 mg/Kg) *in vivo* treatment. Two successive ANOVA test were performed (one on peptides and one on proteins). The quantitative data were only considered at the level of proteins if a minimum of 2 peptides by proteins were quantified. In total, 253 proteins were found dysregulated with a fold change superior or equal at 1.5 at E10.5 and E11.5. Among them, 16 proteins were found dysregulated at both exposure times (upper panel). Among these 253 proteins, 130 have been up-regulated (red) and 117 have been down-regulated (green) and 6 presented an opposite dysregulation between both exposure time (bottom panel).

To examine the time course of molecular changes associated with E9.5 ATRA exposure, we studied embryo head protein levels at E10.5 and E11.5. LC-MS/MS analysis revealed 440 dysregulated proteins and all these proteins were analyzed by the several bioinformatic software (p < 0.05). Among these, 253 were significantly dysregulated at E10 or/and E11, by prenatal ATRA injection with a ratio superior or equal at 1.5 (see detailed statistics in Table S1). Following prenatal ATRA treatment, 110 proteins were significantly dysregulated specifically at E10.5 (upreguated = 62; downregulated = 48) and 127 proteins specifically at E11.5 (upreguated = 65; downregulated = 62) (Fig. 3). Moreover, 16 proteins were dysregulated with a ratio ≥ |1.5| in both exposure times: 3 proteins were upregulated and 7 proteins were downregulated and 6 presented an opposite dysregulation between the both time studied (Fig. 3). (Fig. 3a).

**Figure 4:**
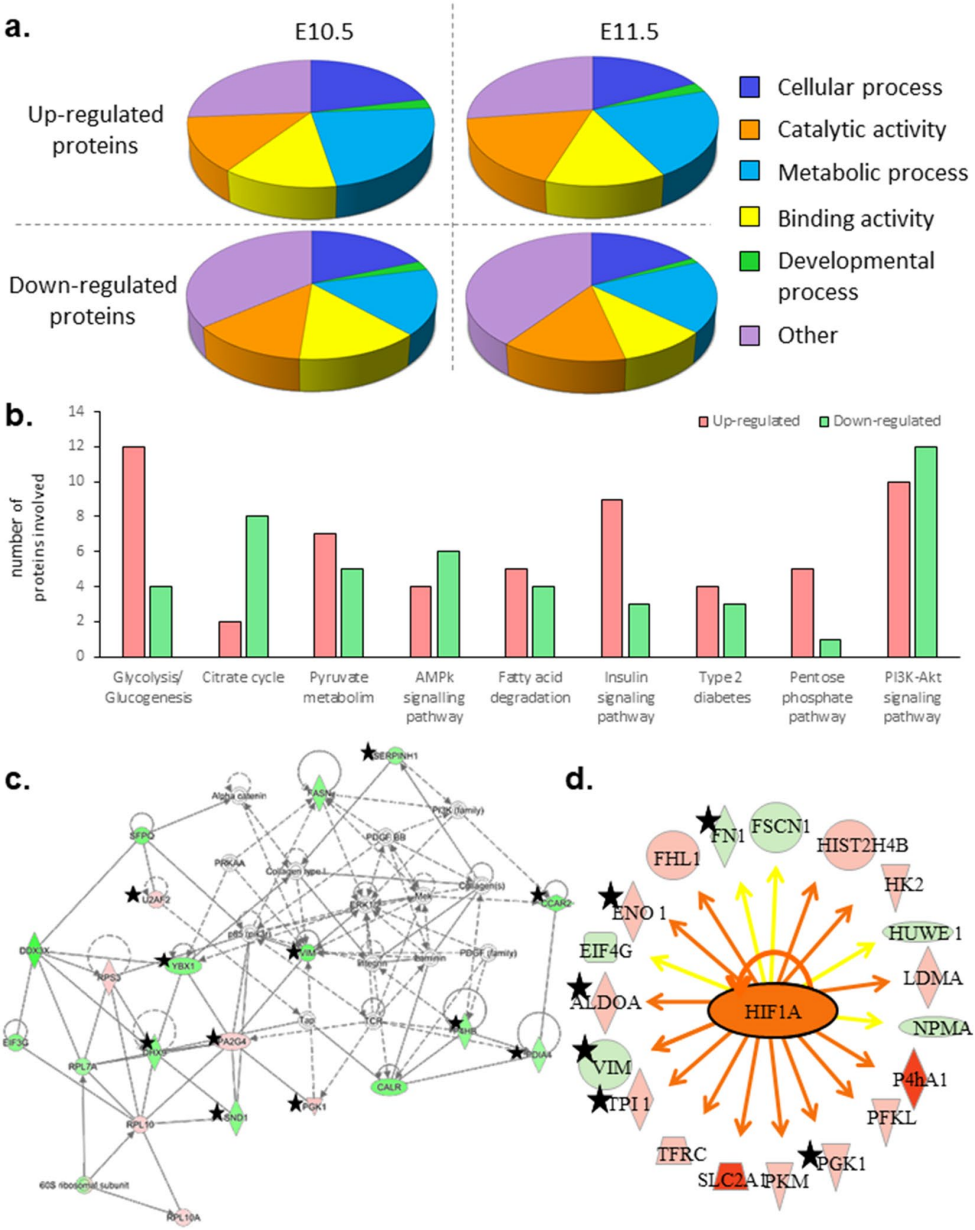
(**a**) Diagrams showing the significant biological processes in which dysregulated proteins were involved, according to Panther classification system. Proteins up and down-regulated have been analyzed separately. Metabolic processes, developmental functions and cellular component organization or biogenesis were enriched in all conditions. (**b**) Histogram presenting the most enriched sub-functions of metabolic processes following STRING software analysis of all dysregulated proteins after 1 and 2 days of prenatal ATRA treatment. 76 dysregulated proteins following ATRA treatment were involved in metabolic processes. Red bars represented the number of up-regulated proteins, the green bars the down-regulated ones. (**c**) «Protein Synthesis, Cell Death and Survival, Cancer» network. According to IPA analysis, it was one of the major network in which proteins dysregulated by antenatal ATRA (after both one and 2 days) are involved. Up-regulated proteins are framed in red and down-regulated proteins in green. (**d**) HIF1A and its network from IPA analysis of proteomics data. Up-regulated proteins are in red and down-regulated proteins in green. In orange, HIF1A is predicted to be activated at E10.5 (1 day of ATRA 100-treatment). The orange arrows referred to activation, whereas yellow arrows evoked an inconsistent finding with state of downstream molecule. The stars highlighted proteins dysregulated in the same way after 1 and 2 days of ATRA-100 treatment.

Significant biological processes were highlighted due to an overrepresentation of involved proteins. Thus, cellular and metabolic processes and also cellular organization or biogenesis components had been enriched, according to Panther analysis (Fig. 4a). In particular, the major Kegg pathways regarding all dysregulated proteins involved the metabolic processes (76 proteins), and were divided into several sub-functions, by String software analysis, where energetic metabolism (Glycolysis/Glycogenesis especially) and insulin signaling pathway were predominant (Fig. 4b).

**Figure 5:**
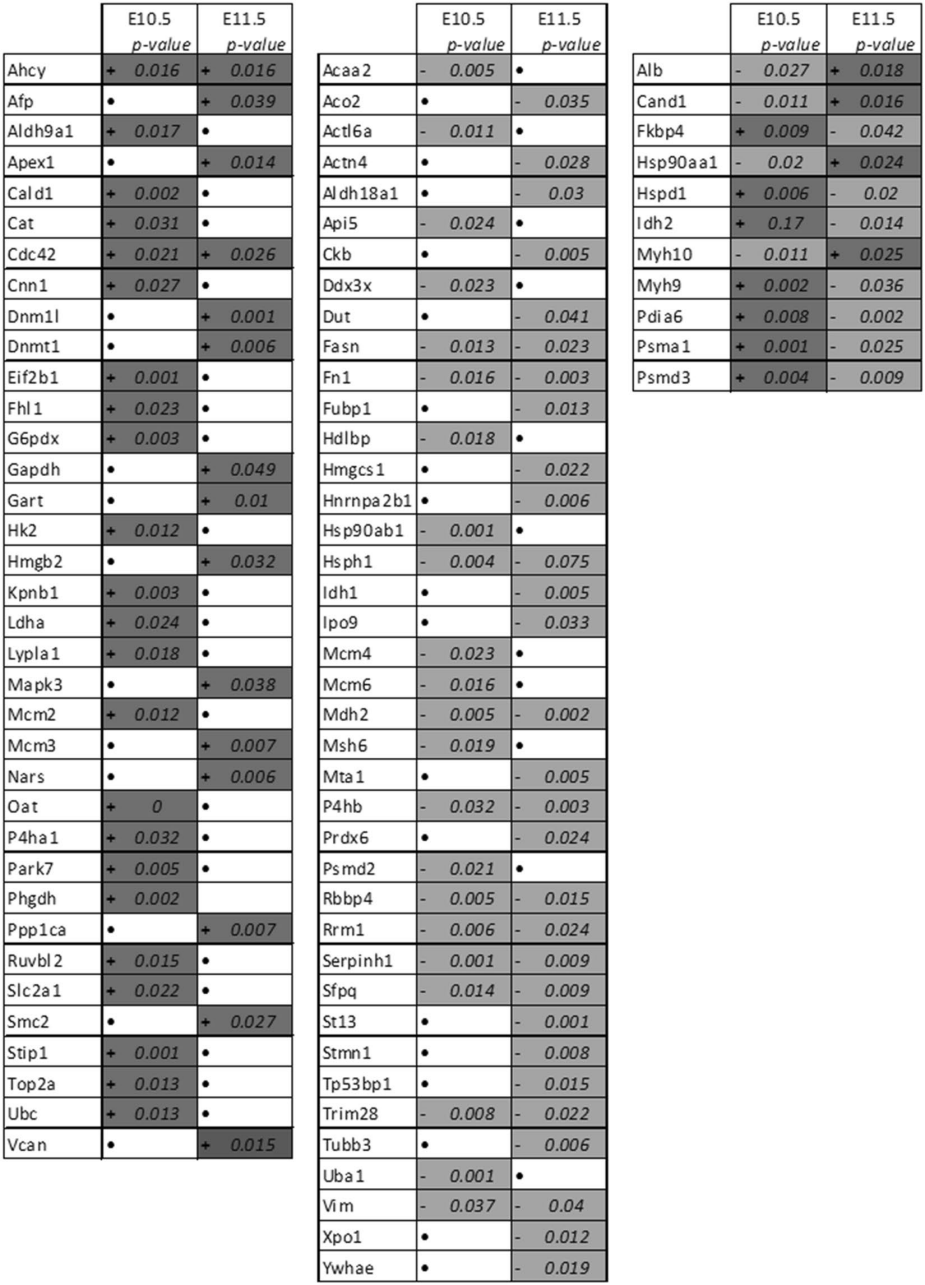
Proteins involved in TP53 pathway (apoptosis) following IPA analysis. Activated (+) or inhibited (−) proteins involved in TP53 pathway have been revealed by IPA analysis. and light-gray boxes represent respectively an increase and a decrease of protein expression observed by proteomics analysis at E10.5 and E11.5 (after 1 and 2 days of ATRA treatment). (FDR < 0.05) No significant variation by LC/MS-MS analysis are represented by •.

Among all pathways highlighted by Ingenuity Pathway Analysis (IPA), those involved in cell death and survival were predominant (Table S2). Indeed, at E10.5, the protein ubiquitination process was one of the “top canonical pathways” affected. Additionally, the TP53 pathway was highly enriched with 34 proteins up-regulated and 46 down-regulated (and 19 differentially regulated) in our experiments (Fig. 5). Thus, cell survival was predicted to be activated at E10.5 and inhibited at E11.5. In contrast, necrosis processes were inhibited at E10.5 and then activated at E11.5. “Protein Synthesis, Cell Death and Survival, Cancer” was one of the major networks altered, according to the IPA analysis, both at E10.5 and E11.5 following ATRA treatment (detailed in Fig. 4c). Moreover, Hypoxia-Inducible Factor (HIF1A) Signaling was considered as one of the major factors in the “Tox List” and HIF1A was predicted to be an activated “upstream regulator” at E10.5 (Fig. 4d).

### ATRA exposure leads to a dysregulation of proteins involved in craniofacial development

**Figure 6:**
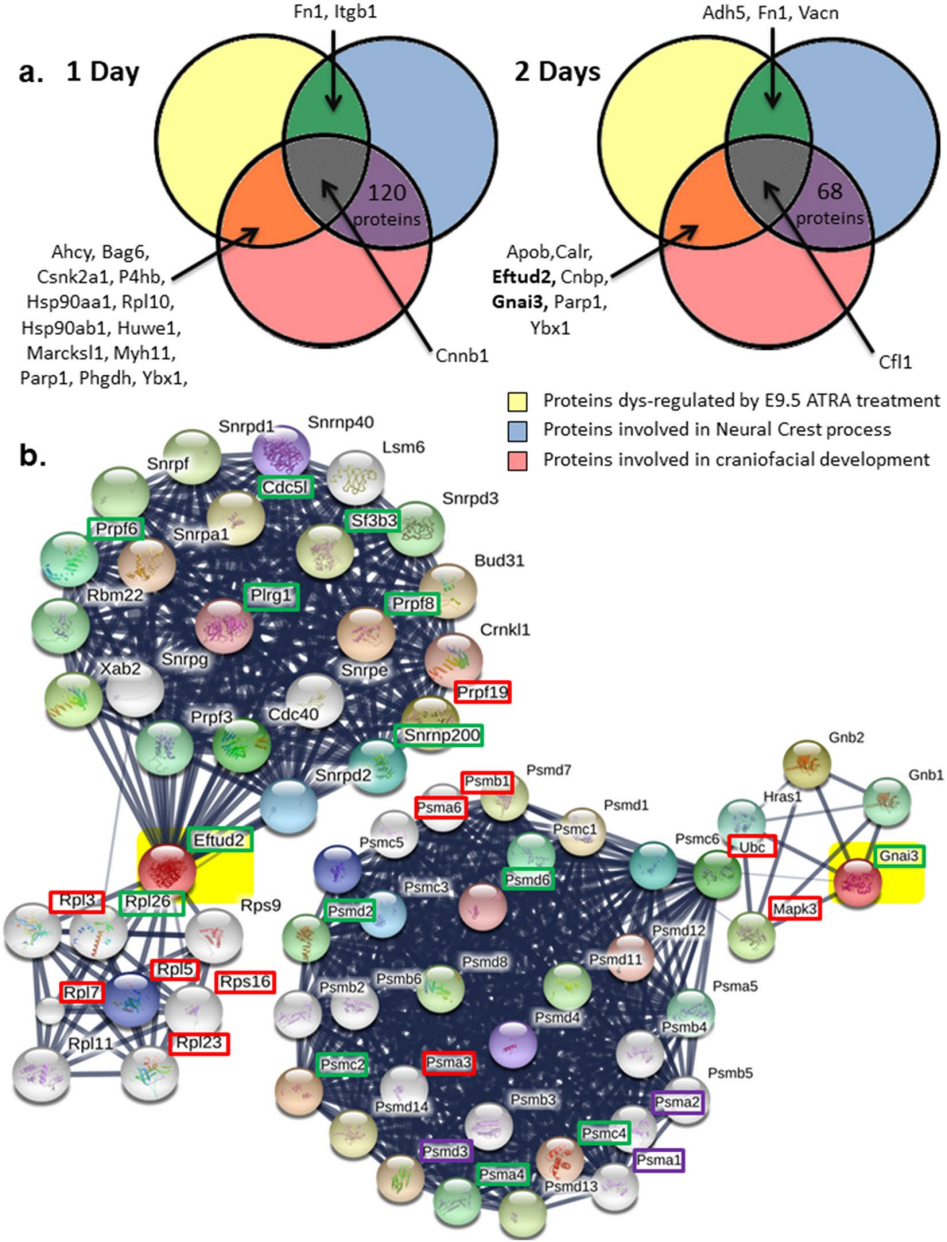
(**a**) Venn diagrams from IPA analysis of regulated proteins by ATRA at E10.5 and E11.5 in our study compared to proteins involved in neural crest process or linked to craniofacial development. Underlined, Fn1, Ybx1 and Parp1 are dysregulated in the same way at E10.5 and E11.5. In bold, Eftud2 and Gnai3 proteins were inhibited at E10.5. These genes are involved in craniofacial disorders: Mandibulofacial dysostosis (Guion-Almeida type) and Auriculocondylar syndrome 1 respectively. (**b**) Eftud2 and Gnai3 STRING network analysis. STRING network revealed protein related to Eftud2 and Gnai3 (highlighted in yellow) proteins based on literature references. Among proteins revealed by this software, some were also found dysregulated in our proteomics data. Thus, framed proteins were found increased (red), decreased (green) or differentially regulated (purple) following 1 and/or 2 days of ATRA treatment.

**Figure 7:**
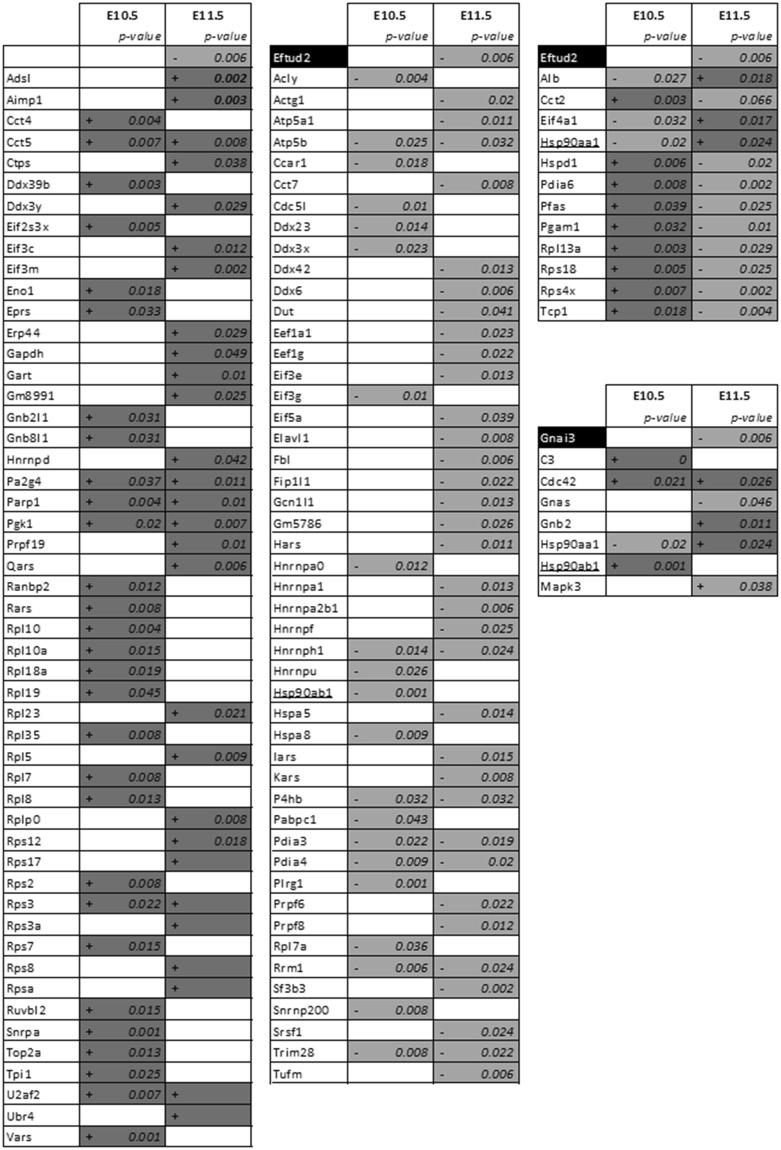
Up and/or down-regulated proteins by ATRA treatment linked with Eftud2 and Gnai3 respectively following STRING software analysis. STRING analysis with *mus musculus* as reference is performed on all dysregulated proteins identified by our proteomics analysis at E10.5 and E11.5 following ATRA treatment at E9.5. Of all the deregulated proteins, those directly linked with Eftud2 (three right panels) and Gnai3 (fourth panel) are reported in this table. Dark-gray (+) and light-gray (**−**) represent respectively an increase and a decrease of protein expression by LC-MS/MS analysis. Hsp90ab1 and Hsp90ab1 underlined were linked to both Eftud2 and Gnai3 following STRING analysis

Our LC-MS/MS and bioinformatics analysis revealed that some dysregulated proteins caused by ATRA treatment are linked to neural crest processes and/or involved in craniofacial development (Figs 4a and 6a). Interestingly, at both E10.5 and E11.5, Fn1, Ybx1 and Parp1 (underlined in Fig. 6a) were three of these genes dysregulated in the same way following antenatal ATRA-treatment (Fig. 6a). Also, Eftud2 and Gnai3 were both down-regulated by about 1.3-fold, after 2 days of ATRA-treatment (E11.5). Looking for interacting partners of Eftud2 or Gnai3 using String software Single Protein by Name/Identifier analysis, several were also found to be dysregulated at both ATRA exposure times (Fig. 6b). Moreover, the String software analysis of all dysregulated proteins in our proteomics data revealed that 147 and 11 of them are directly linked to Eftud2 and Gnai3, respectively, as for example, P4hb, Ddx3x, Rpl26, Rps16 and Sf3b3 linked to Eftud2, plus Cdc42, Gnas, Mapk3, Ubc, and Gnb2 related to Gnai3, as well as Hsp90ab1 and Hsp90aa1 connected to both (Fig. 7).

### *In vitro* ATRA treatments modify target gene expression and disrupt ATDC5 differentiation

**Figure 8:**
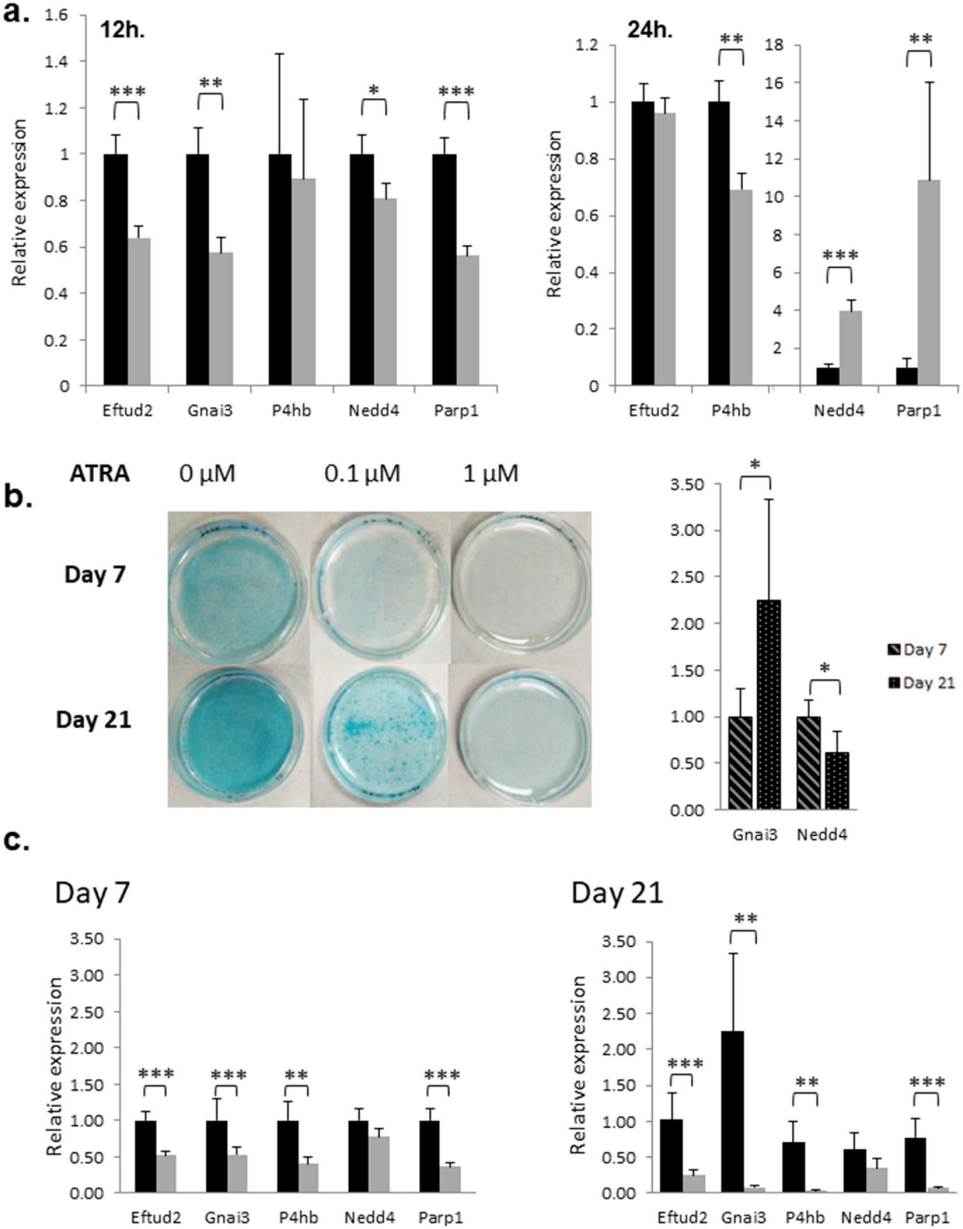
(**a**) *In vitro* RT-qPCR analysis of target genes following ATRA treatment of ATDC5 cells. Relative genes expression of Eftud2, Gnai3, P4hb, Nedd4 and Parp1 following 12 h and 24 h of 1 µM ATRA treatment. Black bars represented control relative mRNA expression although gray bars shown mRNA expression following 1 µM ATRA treatment. (**b**) Peptidoglycans coloration of ATD5 cells by Alcian Blue 7 and 21 days after ATRA treatment and *in vitro* RT-qPCR analysis of targets genes during differentiation. The peptidoglycans accumulation in extra-cellular matrix was used as an index of ATDC5 differentiation into chondrocytes lineage shown by a blue coloration becoming more intense following days. Qualitative analyze suggests an inhibition of ATDC5 differentiation by ATRA. Relative genes expression of Gnai3 and Nedd4 during ATDC5 differentiation, at 7 and 21 days in control condition (no ATRA-treatment). Light turquoise bars represented relative mRNA expression at Day 7 although dark turquoise bars shown mRNA expression at Day 21. (**c**) *In vitro* analysis of target genes expression during ATDC5 differentiation and ATRA chronic treatment. Relative genes expression of Eftud2, Gnai3, P4hb, Nedd4 and Parp1 at Day 7 and Day 21 of ATDC5 differentiation. Impact of 1 µM of ATRA chronic treatment on their expression is also analyzed. Black bars represented control relative mRNA expression although gray bars shown mRNA expression following 1 µM ATRA treatment. Quantitative expression of studied genes was determined using the 2−ΔΔCt method. Statistical significance was evaluated using t-test (*P < 0.05; **P < 0.01; ***P < 0.001; n = 4).

Firstly, expression analysis of 32 selected genes was performed by RT-qPCR, on RNA extracted from E10.5 embryo heads, in order to compare with proteomics data obtained on E10.5 or E11.5 embryo heads. These genes were usually expressed in the same way between proteomics and transcriptomics (Table S3). Secondly, ATDC5 cells treated by 1 µM of ATRA during 12 h or 24 h revealed a significant dysregulation of 8 and 9 genes, respectively. Among them, *Eftud2*, *Gnai3*, *Nedd4* and *Parp1* were significantly down-regulated (respectively, 1.56, 1.74, 1.24 and 1.78 as fold changes; all p at least < 0.05) following 12 h of treatment; *Rarb was* used as a positive control. After 24 h of treatment, *P4hb* expression decreased by a 1.44 factor whereas *Nedd4* and *Parp1* expression increased about 4-fold (p < 0.01) and 10-fold (p < 0.001) respectively (Table S3 and Fig. 8a).

Then, we performed experiments to differentiate ATDC5 into the chondrocytes lineage. This differentiation was attested by the Alcian Blue coloration of the peptidoglycan accumulation in the extracellular matrix. ATRA chronic treatment at 0.1 and 1 µM drastically inhibited ATDC5 differentiation in a dose-dependent manner (Fig. 8b). RNA was extracted from cultured cells for each condition and quantified without finding any difference between RA-treated cells and control cells (data not shown). Some targets highlighted *in vitro* were analyzed. In the control condition (no ATRA added), a 2.25-fold increase of *Gnai3* expression was observed during differentiation whereas *Nedd4* expression decreased with a 1.65 ratio (Fig. 8b). Moreover, *Eftud2*, *Gnai3*, *P4hb*, *Parp1* were significantly repressed by 1 µM ATRA treatment after 7 days of differentiation with around a 2-fold ratio (1.9; 1.9; 2.4 and 2.7-fold change ratio respectively; p at least < 0.01). Interestingly, after 21 days of differentiation the expression of these genes is even more reduced with a decreased ratio of 4.0; 28.1; 17.5 and 10.9 in cells treated by 1 µM ATRA compared to the control cells (p at least < 0.01) (Fig. 8c).

## Discussion

RA, the major active vitamin A derivative, is essential for proper embryonic development. Both increased or decreased RA signaling could induce developmental syndromes with craniofacial abnormalities as shown in patients carrying *CYP26B1*^40^, *RARB*^41,42^ or *STRA6*^43^ mutations and in a rodent model where *Cyp26b1*^−/−^ mice presented craniofacial features^44^. Additionally, ATRA exposure and RA-deficiency are closely linked by a rapid change in expression levels of enzymes involved in ATRA metabolism^25^. Due to its ability to regulate gene expression and the involvement of RA-related genes in developmental syndromes with craniofacial features, we focused our study on the link between craniofacial disorders, such as OAVS, and ATRA in order to identify new candidate genes for these syndromes. Thereby, mutations in genes dysregulated by ATRA, during a key window of craniofacial development, should be potentially a cause of craniofacial syndromes. The first two branchial arches, the neural tube, the stomodeum, the eyes and the newly differentiated ears appear at E9.5 in mice, thus corresponding to an appropriate developmental window to study the impact of ATRA exposure on craniofacial development. The high embryos’ resorption revealed potential unspecific effects associated with prenatal ATRA treatment above the linear dose-response phase and this mortality might increase the risk of revealing unspecific proteins. Indeed, the presence of dysregulated proteins related to organismal injury, gastrointestinal disease and renal damage (Table S2) argued for unspecific pathways regarding craniofacial development. However, these data showed probable visceral defects, already evoked in the literature^45^, and illustrated the multiple damaging effects observed following gestational RA exposure^18^. However, Studying the length of Meckel’s cartilage of treated embryos led to the identification of specific craniofacial defects following ATRA 100 treatment and thus confirmed the adequate developmental stage to link ATRA to craniofacial defects (Fig. 2). Despite the strong phenotype observed in one embryo treated with 50 mg/kg, (Fig. 1d), no statistical difference was observed between ATRA-50 and control embryos (Fig. 2). Thus, we chose to focus our molecular study on embryos treated with 100 mg/kg.

Proteomic studies are a global approach that allowed us to identify new ATRA targets in an *in vivo* context. To be more accurate and to enrich our samples with proteins potentially involved in craniofacial development, only proteins extracted from the head and the neck of embryos were analyzed. Among the 553 dysregulated proteins identified by LC-MS/MS in treated embryos, following 1 or 2 days of treatment, some proteins were involved in RA signaling, such as Crabp1 (Cellular Retinoic Acid Binding Protein), and Parp1 (Poly(ADP-ribose) polymerase 1) which is an important cofactor for gene expression induction by RA via RARB^46^. Moreover, not only RAR activation but also PPAR/RXR activation, another nuclear receptor complex linked to RA signaling pathway by the common RXR heterodimer^47^, was highlighted by IPA as one of the major “Tox list” hits from proteins commonly regulated at E10.5 and E11.5 following E9.5 ATRA treatment (Table S2).

Currently, several types of software can be used to analyze proteomics data, and their combination seems important to get a robust overview of the disrupted pathways or the cell functions affected by ATRA treatment. Thus, bioinformatics analysis has been performed with IPA, String software and Panther, and we found that the data obtained highlighted similar functions. For example, proteins involved in protein synthesis (Table S2 and Fig. 4d) were well represented among the total of dysregulated proteins caused by ATRA treatment. Accordingly, to change the expression of some ATRA targets, the protein synthesis function should be activated. Due to the overrepresentation of proteins involved in protein synthesis (Table S1, Fig. 4d), but also with the high number of ubiquitination factors highlighted in our data (Table S1, Figure S1A), both translational and post-translational regulation might occur in parallel.

Herein, we report that ATRA treatment might alter cellular metabolic networks (Fig. 4a,b). In particular, glycolysis/glucogenesis and insulin signaling pathways were two among the top altered canonical pathways following IPA analysis with around 18% (14/76) and 14% (11/76) of proteins involved in related metabolic pathways, respectively (Table S2, Figure S1B). Additionally, the Insulin receptor (Insr) pathway was predicted by IPA to be inhibited at E10.5. Interestingly, maternal diabetes is associated with an higher risk of OAVS features^12^, and Mapk3, Pkm and Hk2-genes related to type II diabetes^48^ were upregulated by ATRA in our *in vivo* conditions, suggesting a clue for a possible crosslink between ATRA signaling, diabetes and craniofacial disorders. Additionally, diabetes and RA signaling are already associated with STRA6, a retinol transporter also implicated in a developmental disorder involving craniofacial features^43^. Of note, STRA6 has been shown to inhibit insulin receptor responses in white adipose tissue^49^.

Moreover, several proteins involved in anaerobic metabolism such as AldoA, Slc2a1, Eno1, Pkm have been found up-regulated in our treated samples. Hypoxia-inducible factor 1-alpha (HIF-1A) is a major coordinator to induce the shift between aerobic (oxidative phosphorylation) and anaerobic (glycolytic) metabolism^50^ under low oxygen level conditions. Hypoxia factor signaling was highlighted in the “Tox list” at E10.5 (Table S2) and Hif1a was predicted to be activated (fold change +2.2) at E11.5 (Fig. 4c). We note that Hif1a expression itself was not dysregulated, probably due to a post-translational regulation^51^. Additionally, the close link between ATRA and Hif1a was reinforced by the down-regulation of Hif1a observed in mice treated by a RAR antagonist^52^. Interestingly, a few dysregulated proteins related to Hif1a (Fig. 4c) were also shown to be involved in embryonic development and especially, in neural crest processes (Figs 4c and 5a); in particular, Fn1 whose role in cell differentiation and migration is major for proper vertebrate embryonic development^53^, P4hb which is involved in Cole-Carpenter syndrome (MIM: #112240) characterized by bone fragility, craniosynostosis, ocular proptosis, hydrocephalus, plus other facial defects^54^, and Cdc42 which when knocked out in neural crest cells leads to facial cleft or other craniofacial abnormalities^55^. Among other upstream regulators revealed by IPA, F2 or Prothrombin was predicted as inhibited in E11.5 embryo heads and its pathway shared some common factors with Hif1a signaling (Figure S1C). We note that F2 is involved in wound healing and is associated with thrombophilia and susceptibility to miscarriages (MIM: #614390)^56^. Taking together, our data point out a molecular link between antenatal ATRA-exposure and hypoxia or vascular disruption, another environmental factor related to OAVS^14,15^.

Furthermore, a crucial role of Hif1a has been shown to permit cell adaptation under certain environmental conditions, thus allowing cell survival^57^. As shown for the Hif1a pathway, cell survival was also predicted to be activated after 1 day of ATRA-treatment (E10.5). Then, a molecular shift seemed to appear to activate apoptosis/necrosis mechanisms, 2 days after ATRA exposure (E11.5). Thus, we observed, here, a precise regulation of expression of proteins involved in these pathways which does not support an unspecific over-representation of apoptosis network due to the high embryonic lethality. Indeed «Protein Synthesis, Cell Death and Survival, Cancer» (Fig. 4d) and the Tp53 pathway (Fig. 5) supported the relevance of apoptotic mechanisms in this teratogenic context. In addition, the ubiquitin system, particularly involved in protein degradation, was highly represented (Figure S1A) with Nedd4 which when knocked out presents craniofacial defects linked to an increase of apoptosis of neural crest cells^58^. Keeping in mind that pathogenic mechanisms involved in Treacher Collins syndrome are dependent on the Tp53 pathway^4^, our *in vivo* ATRA-treatment results show similar processes where ATRA-exposure led to an increase of apoptosis processes at late time points and thus could participate in producing the developmental defects observed.

The identification of dysregulated proteins involved in neural crest processes or more widely in craniofacial development (Fig. 6a) seems relevant for potential new candidate genes for craniofacial disorders regarding their role during embryogenesis. For example, the role of Parp1 in craniofacial development was revealed by IPA and could be an excellent candidate, all the more since neural crest, ear,and pectoral fin defects are observed after *parp3* inactivation in zebrafish^59^.

**Figure 9:**
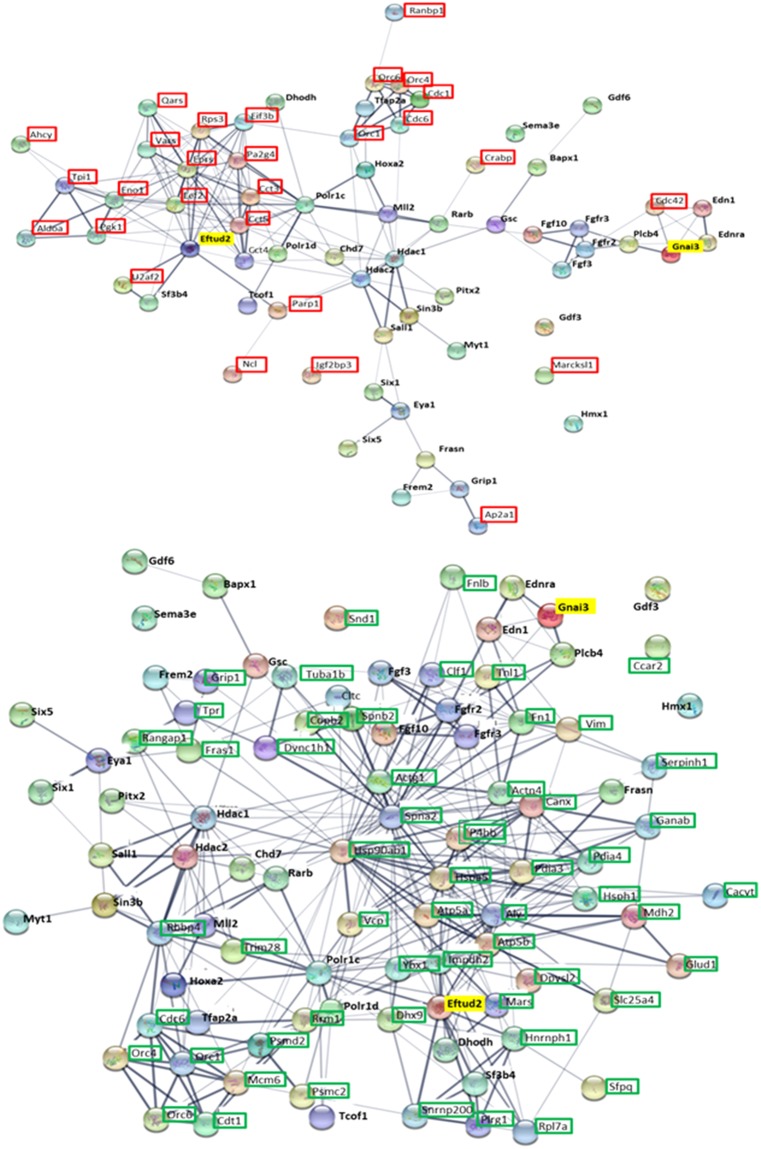
Interactions between proteins whose selected genes^6^ are involved in craniofacial developmental diseases and proteins found dysregulated following ATRA treatment (both 1 and 2 days). The Networks were obtained by STRING network analysis. Proteins framed in red are up-regulated (up panel) whereas proteins down-regulated are framed in green (down-panel), at both experimental times. Gnai3 and Eftud2, involved in craniofacial syndromes were also found repressed after 2 days of ATRA treatment (highlighted in yellow).

To get another view of the link between ATRA-target proteins and craniofacial development, we connected proteins whose expression was dysregulated in the same way at both E10.5 and E11.5 following an *in vivo* antenatal E9.5 ATRA-injection with proteins related to craniofacial disorders already characterized^6^ (Table S4). The String network thus obtained (Fig. 9) revealed interactions among these proteins such as *P4hb or Actg1* which are related to syndromes presenting craniofacial features (Table S3). This connection highlights their potential contribution to the defects observed in the ATRA-treated embryos and so, by extrapolation, propels the targets associated in this network as potential good candidate genes for craniofacial disorders. In addition, our experimental data revealed a down-regulation of Eftud2 and Gnai3 in the head of E11.5 embryos (following 2 days of antenatal ATRA treatment), and these genes are involved in Mandibulofacial dysostosis, Guion-Almeida type (MIM: # 610536) and Auriculocondylar syndrome (MIM: # 602483) respectively. Moreover, close Eftud2 and Gnai3 partners were also found dysregulated in our samples arguing for their potential role in the observed craniofacial defects. Thus, our study using both proteomic and transcriptomic approaches, reveals for the first time a link between ATRA and *Eftud2* and *Gnai3*.

To study further the dysregulation of the RA-targets, we examined transcriptomic regulation of some of them in E10.5 treated-embryos (Table S3). We observed a quite good correlation between RNA and protein *in vivo* data and also with *in vitro* analysis following 12 and 24 h of ATRA-treatment of ATDC5 cells. Among all, targets both regulated at RNA and protein level may refer to a stronger relevance. To link ATRA *in vitro* cell treatment to craniofacial cartilage defects observed in our *in vivo* experiments, we performed ATDC5 differentiation into the chondrocyte lineage, and focused on the expression of several genes revealed by our *in vivo* approach. The variation of gene expression observed for some targets due to ATRA-treatment might explain the drastic perturbation of ATDC5 differentiation^60^ (Figs 8b and 6c). In particular, *Gnai3* was up-regulated during ATDC5 differentiation between day 7 and day 21 but chronic ATRA-treatment led to a drastic down-regulation of this gene. Thus, this dysregulation could participate in the decrease of cell differentiation and, by extrapolation to our *in vivo* result, could effect craniofacial development and led to craniofacial abnormalities.

In conclusion, we have combined an *in vivo* approach by using ATRA as a teratogenic agent and an *in vitro* model of cell differentiation to better understand molecular pathways involved in RA signaling during craniofacial development. Our data suggested a probable common mechanism of several environmental factors causing craniofacial syndromes. We note that the molecular links between RA, hypoxia, and diabetes revealed in our study reflect those shown between the RA signaling pathway and cigarette smoking^61–63^ or ethanol exposure^64–66^ which are other environmental factors associated with embryonic defects. Finally, our models offer new target genes for craniofacial disorders and thus help to decipher molecular pathways involved in craniofacial development. The dysregulation of Gnai3 and Eftud2 genes, involved in known syndromes associated with craniofacial features, reinforced the relevance of our data in order to identify genes linked with abnormal craniofacial development. Thus, future studies should study these targets among cohorts of patients to identify pathogenic nucleotide variants leading to the phenotype.

## Material and Methods

### Animal and ATRA treatment

Gestant C57Bl/6NRj female mice (Janvier Lab, Le Genest St Isle, France) were maintained in a 12-h light/12-h dark cycle (lights on at 08.00 hours) in a temperature-controlled room (22 °C) with free access to food and water. Intraperitoneal injection of ATRA 50 mg/Kg, 100 mg/Kg or DMSO (control) was injected performed on time mating mice at E9.5. Dams were sacrificed by a lethal dose of pentobarbital (250 mg/kg), a midline incision was made down the ventrum and the uterine horns were exposed in order to collect embryos for expression (E10.5 or E11.5) and phenotypic analysis (E14.5).

### Alcian Blue and Alizarin Red Coloration

E14.5 embryos were washed with PBS. Then, they were fixed in Ethanol (EtOH) 95% (2 days), dehydrated by acetone 100% (2 days) and colored, during 5 days at 40 °C with gentle agitation, with Alcian Blue and Alizarin Red solution (150 µg/mL Alcian Blue, 50 µg/mL Alizarin Red dissolved in 67.75% EtOH and glacial acetic acid 5%). Successive washes were performed (EtOH70%, 50%, 25%, then ddH_2_O) preceded discoloration by KOH 0.5% during 24 h to reveal only specific coloration The embryos were then incubated in the following solutions: KOH 0.5% - Glycerol 20% 7 days, KOH 0.2%-Glycerol 50% 4 days, KOH 0.2%-Glycerol 80% 4 days). Embryos were stored in 100% glycerol at 4 °C.

### Proteomics

#### Sample preparation and protein digestion for LC-MS/MS analysis

Proteins Extraction from the head of embryos at E10.5 (1 Day of treatment) and E10.5 (2 Days of treatment) was performed using RIPA Buffer (SigmaAldrich) and Proteases/phosphatases inhibitor (Sigma Aldrich) with sonication (30 min, 30 s/30 s, Amplification 65%). Each sample was denatured, solubilized in Laemmli buffer (Biorad) and separated on 10% SDS-PAGE. The gel was stained with colloidal Blue (Biorad) and SDS-PAGE lanes were cut in 1 × 1 mm gel pieces, treated with destaining solution (25 mM ammonium bicarbonate and 50% acetonitrile (ACN)) and then rinsed twice in ultrapure water and shrunk in ACN for 10 min. After ACN removal, gel pieces were dried at room temperature (RT), covered with trypsin solution (10 ng/µl in 40 mM NH_4_HCO_3_ and 10% ACN), rehydrated at 4 °C (10 min), and finally incubated overnight at 37 °C. They were incubated in 40 mM NH_4_HCO_3_ and 10% ACN at RT (15 min, with rotary shaking). The supernatant was collected, and an H_2_O/ACN/HCOOH (47.5/47.5/5, v/v/v) extraction solution was added onto gel slices (15 min, repeated twice). Supernatants were pooled and concentrated in a vacuum centrifuge to a final volume of 25 µL. Digests were finally acidified by 1.5 µL of formic acid (5%, v/v) and stored at −20 °C.

#### LC-MS/MS analysis

The peptide mixture was analyzed on a Ultimate 3000 nanoLC system (Dionex, Amsterdam, The Netherlands) coupled to an Electrospray LTQ-Orbitrap XL mass spectrometer (Thermo Fisher Scientific, San Jose, CA). Ten microliters of peptide digests were loaded onto a 300-µm-inner diameter x 5-mm C_18_ PepMap^TM^ trap column (LC Packings). The peptides were eluted from the trap column onto an analytical 75 mm id x 15 cm C18 Pep-Map™ column (LC Packings) with a 5–40% linear gradient of 0.1% formic acid in 80% ACN. The separation flow rate was set at 300 nL/min. The mass spectrometers operated in positive ion mode at a 1.8 kV needle voltage. Data were acquired in a data-dependent mode. The scans were recorded with a resolution of R = 70,000 (@m/z 200) in MS and R = 35,000 n MS/MS. Top 15 ions were selected from fragmentation in MS/MS mode. Additionally, dynamic exclusion was set to 30 s and only doubly or triply charged are selected for fragmentation.

#### Database search and results processing

Data were searched by SEQUEST through Proteome Discoverer 1.4 (Thermo Fisher Scientific Inc.) against a subset of the UniProt database, limited to the *Mus Musculus* Reference Proteome Set. The spectra of peptides higher than 5 kDa or lower than 350 Da were rejected. The search parameters were as follows: the mass tolerance of the precursor ions and the fragment ions is set at 10 ppm and 0.02 Da respectively. Only ions b- and y-ions are considered. Oxidation of methionines (+16 Da) was considered as a variable modification and the carbamidomethylation of cysteines (+57 Da) as a fixed modification. Two missed trypsin cleavages are allowed.

Validation of the peptides was carried out using the “Percolator” algorithm and only the “high confidence” peptides are retained, which corresponds, at the peptide level, to a false positive rate of 1%.

### Bioinformatics software

Data (all 440 dysregulated proteins) were analyzed through the use of IPA (QIAGEN Inc., https://www.qiagenbioinformatics.com/products/ingenuity-pathway-analysis), *STRING* database (http://string-db.org), and Panther software (http://www.pantherdb.org/panther/summaryStats.jsp).

### RNA Extraction and RT-qPCR

ARNs from embryo and cells were extracting using RNeasy Plus Mini/micro kit extraction (Qiagen) following Qiazol-Chloroform lyse. Reverse transcriptions were performed using MulV reverse transcriptase (Thermo Fisher) with 1 µg of RNA as a matrice. Quantitative PCR (qPCR) was performed by using the iQTM SYBR Green Supermix and Icycler CFX96 real time-PCR detection system (Bio-Rad). Quantitative expression of studied genes was determined using the 2^−ΔΔ^Ct method with *Ppia* and *Hprt* as reference genes; primers used are listed in Table S5.

### Cell culture

ATDC5 cells (mouse prechondrocytes) were kindly provided by Dr. Guicheux (UFR Odontologie-UMR 791. LIOAD (Nantes)). The cells were maintained in DMEM/F-12, GlutaMAX™(Gibco Life technologies), supplemented by 5% fetal bovine serum and 1% Penicillin-Streptomicin 1% in a humidified incubator at 37 °C, with 5% CO_2_. ATDC5 were differentiated into chondrocytes using the same medium as maintenance, without serum and complemented with Insulin-transferrin-sodium selenite (ITS bovin Insulin and human transferrin 25 µg/mL, sodium selenite 25 ng/mL; Sigma Aldrich, ref. I1884) dissolved in 0.1% acetic acid. ATDC5 cells were treated with 1, 0.1 µM or DMSO (control) during 12 or 24 h. During ATDC5 differentiation, ATRA was added every 2 days (during medium replacement) since Day 0. Cells were washed by PBS, fixed by 4% PFA (10 minutes) and then colored overnight under agitation with Alcian Blue and Alizarin red solution. Several washes were performed with ddH_2_O and the cells were stored at 4 °C.

### Statistical Analysis

All data are expressed as mean ± SEM. For all analyses, differences were considered significant at p < 0.05.

#### Embryos morphological analysis

Groups comparison for craniofacial cartilage size and angle measures were analyzed by ANOVA followed by Tukey post hoc tests (GraphPad Software, San Diego, California, USA).

#### Proteomics analysis: Quantitative data analysis without labeling

The raw LC-MS/MS data is imported into Progenesis LC-MS 4.1 (Nonlinear Dynamics Ltd, Newcastle, U.K). The processing of the data comprises the following steps: 1/detection of ions, 2/alignment of the ions through the whole set of data, 3/integration of the volumes for the ions whose state of charge ranges from 2 to 6, 4/standardization, 5/importing sequence information, 6/- ANOVA test at peptide level and filtering for p-value < 0.05, 7/calculation of the abundance of the proteins (sum of the volume of the 3 major corresponding peptides), 8/- ANOVA test at the protein level and filtering for p-value < 0.05. Only non-confrontational and unique peptides are considered for calculation at the protein level. The quantitative data are only considered at the level of proteins if a minimum of 2 peptides is quantified.

#### qPCR analysis

Student t tests were used to compare gene expression between control and ATRA treated groups, both *in vitro on ATDC5 cells* and *in vivo* embryos experiments by CFX Manager (Biorad, France).

### Study approval

All experiments were approved by the Bioethical committee of the University of Bordeaux (N° 50120186-A) and région Aquitaine Veterinary Services (Direction Départementale de la Protection des Animaux, approval ID: A33-063-920) according to the European (Directive 2010/63/EU, 2010 September 22th) legislation.

## Electronic supplementary material

Figure S1

Table S1

Table S2

Table S3

Table S4

Table S5
