# Supplementary material for: Prenatal retinoic acid exposure reveals candidate genes for craniofacial disorders
Source: Sci Rep. 2018 Nov 30;8:17492. doi: 10.1038/s41598-018-35681-0 (PMC6269437; doi:10.1038/s41598-018-35681-0)
Supplement: Supplementary file 1 — Figure S1 [file 41598_2018_35681_MOESM1_ESM.pdf]

Prenatal retinoic acid exposure reveals candidate genes for craniofacial disorders

Marie Berenguer[1], Muriel Darnaudery[2], Stéphane Claverol[3], Marc Bonneu[3], Didier Lacombe[1,4], Caroline Rooryck[1,4,*].

[1] Univ. Bordeaux, Maladies Rares: Génétique et Métabolisme (MRGM), U 1211 INSERM, F-33000 Bordeaux, France

[2] Université de Bordeaux, Nutrition et neurobiologie intégrée (NUTRINEURO), UMR 1286, 146, rue Léo Saignat, 33076 Bordeaux Cedex, France - Inra, Nutrition et neurobiologie intégrée (NUTRINEURO), UMR 1286, F-33076 Bordeaux, France.

3 Center of Functional Genomics, Bordeaux University, Bordeaux, France.

[4] CHU de Bordeaux, Service de Génétique Médicale, Centre de Référence Anomalies du Développement et Syndromes Malformatifs, F-33000, Bordeaux, France

Supplemental Figure S1

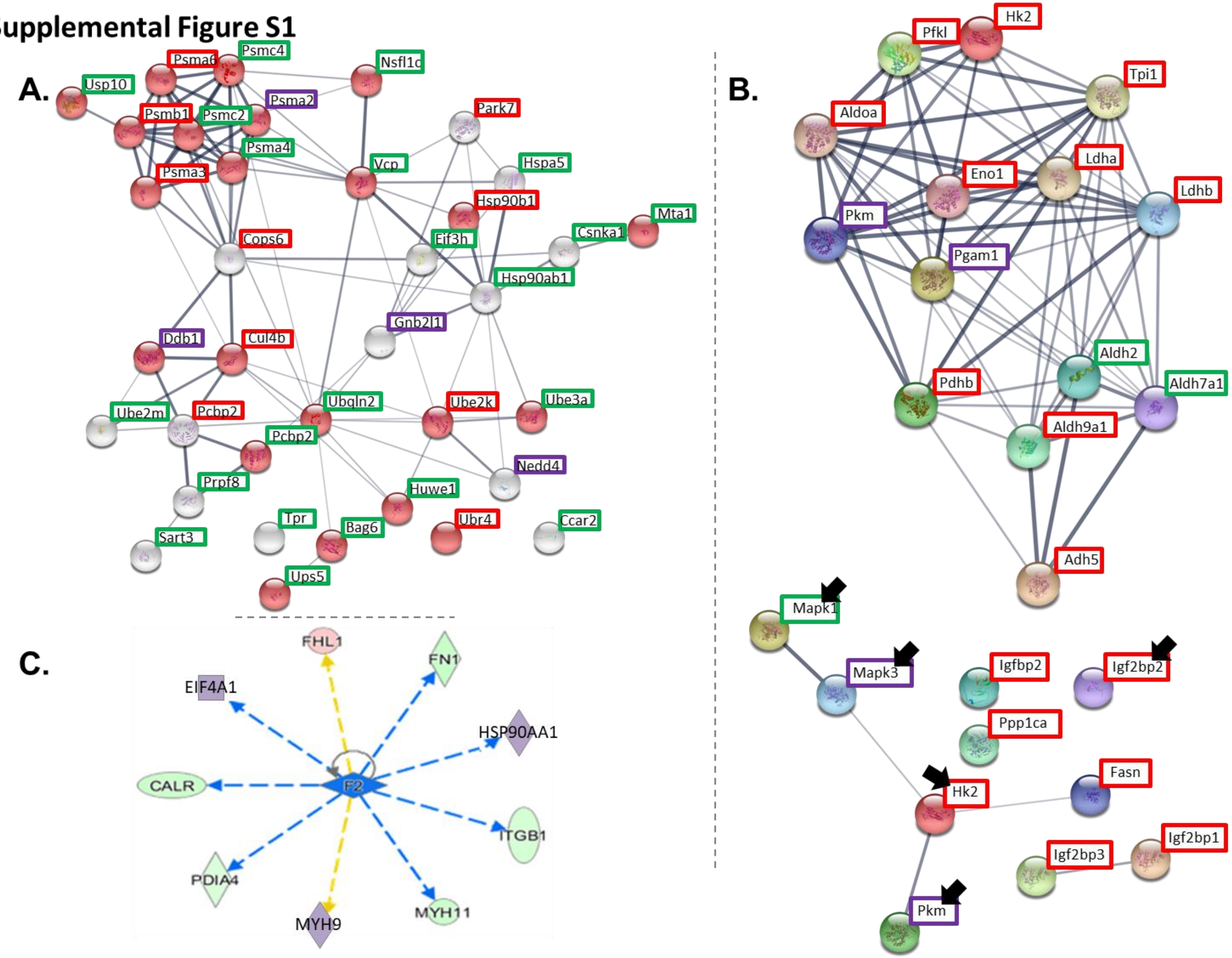

Supplemental Figure S1

A. STRING® network of the 36 proteins involved in ubiquitination system, following the analysis of all dysregulated proteins (1 and 2 days of treatment). Among them, proteins involved in ubiquitin-dependent protein catabolic process are colored in red.

B. STRING® network of the 14 proteins involved in Glycolysis and Glycogenogenesis by STING® Software (up panel). All dysregulated proteins (1 and 2 days of treatment) were analyzed. Glycolysis/Glycogenesis is one of the major Kegg pathways relative to metabolic processes in our samples.
STRING® network of the 10 proteins involved in Insulin signaling pathway (down panel). Arrows highlighted proteins also linked to type II diabetes. All dysregulated proteins (1 and 2 days of treatment) were analyzed.

C. Network of F2/ Prothrombin, considered by IPA® analysis as an inhibited Upstream regulator following both 1 and 2 days of *in vivo* ATRA-treatment.

Proteins are up-regulated are framed in red, down-regulated in green following 1 and/or 2 days of ATRA-treatment, in purple, proteins are differentially regulated between 1 and 2 days of *in vivo* treatment.
